# Laughter in the making: social bonding and coordination in dance practices

**DOI:** 10.3389/fpsyg.2026.1754425

**Published:** 2026-06-23

**Authors:** Finn Upham, Bilge Serdar, Alexander Refsum Jensenius

**Affiliations:** Department of Musicology, RITMO Centre for Interdisciplinary Studies in Rhythm, Time and Motion, University of Oslo, Oslo, Norway

**Keywords:** cooperation, coordination, dance, dance learning, heart rate, interaction, laughter

## Abstract

Laughter is a ubiquitous human behavior with distinct social functions and evolutionary value. And yet its role in artistic and cultural practices has rarely been investigated, except in explicitly humorous performances. This study highlights laughter's central role in the participatory and developmental phases of dance. Grounded in social function frameworks, we argue that laughter functions as a vital mechanism of layered coordination, supporting group dynamics beyond verbal interaction. Through a multimodal analysis of three distinct dance contexts—an amateur line dancing lesson, a Norwegian folk dancing session, and professional duo improvisations (co-located and remote)—we investigate how and why laughter emerges in real-time creative and learning processes. Using video, physiological (heart rate, core body motion), and phenomenological data, we show that laughter is not a break in action but an integral, embodied tool for coordination development. Our exploratory study demonstrates that laughter serves to acknowledge mistakes, negotiate decisions, express difficulty, and share joyful success in dance. Critically, the quality and shared nature of laughter instances were shaped by the available communication channels, the modalities involved, and the direction of interaction. We conclude that laughter is a powerful, efficient form of embodied communication that helps dancers navigate uncertainty, build interpersonal synchrony, and co-construct shared understanding. These results underscore the importance of studying the evolutionary and social value of artistic practices as lived, co-created experiences beyond their most professional and elite forms.

## 1 Introduction

Across human cultures, the arts have long served as central mechanisms for social cohesion, cooperation, and emotional bonding (Cross, 2001; Grau, 2016; Savage et al., 2021). Within this broader landscape, activities such as dance and music function as embodied social glue across cultures, bringing people together through coordinated movement, shared rhythm, and mutual effort. Dance, in particular, is widely recognized for its positive impact on pain thresholds (Tarr et al., 2015), feelings of unity, belonging and cooperation (Reddish, 2012; Reddish et al., 2013), and the formation of group coalitions (Hagen and Bryant, 2003). Evolutionary perspectives suggest that synchronized and exertive co-movement stimulates endorphin release, thereby reinforcing social bonds among participants (Tarr et al., 2017; Tarr and Dunbar, 2024). These bonding effects are not limited to staged performances but emerge most powerfully in participatory contexts–rituals, festivals, communal gatherings, and dance classes–where individuals act with shared intention and mutual engagement.

Laughter is another behavioral mechanism that plays a key role in human bonding. From an evolutionary perspective, laughter has been described as a form of “vocal grooming,” allowing individuals to “groom at a distance” and maintain social cohesion in larger groups by triggering endorphin release (Dunbar et al., 2021; Dunbar, 2022). Like coordinated movement, laughter affects group affiliation, regulates group dynamics (Glenn, 2003; Wood, 2018), and contributes to the playful (Grant-Jacob, 2024; Holt, 2016), exploratory dimensions of social life (Bryant, 2020). It is also closely connected to learning, exploration, and the development of shared understanding across species (Davila-Ross et al., 2011). Despite these shared evolutionary roots and overlapping social functions, laughter has received remarkably little attention in dance studies. One of the few contributions from an evolutionary angle explicitly frames dance as “grooming at a distance,” paralleling the social functions of laughter and dance (Tarr et al., 2017).

The question of laughter's role in dance contexts arose directly from observing amateur dancers' heart rates during a line dancing lesson. The strongest factor on dancers' heart rates should be the work of energetic movement; participatory dance activities also include social and cognitive demands that impact heart rate from moment to moment. Performance stress and cognitive challenges generally induce increased heart rate in more controlled conditions (Sequeira et al., 2021; Rösler et al., 2021; Iffland et al., 2014), while experiences like social rejection can produce short-term decreases characteristic of parasympathetic activation (Gunther Moor et al., 2010). Participants in this line dancing lesson showed rapid increases in heart rates coinciding with bursts of laughter across the group, suggesting this shared behavior would also be a significant contributor to any measure of physiological synchrony. Considering laughter in response to humorous stimulus is widely documented to lower heart rate post-stimulus (Law et al., 2018; Bains et al., 2017; Averill, 1969), the observed pattern motivated a closer look at the context. A review of the session recordings exposed laughter throughout the lesson, often arising without intentionally humorous prompts. The prominence and impacts of laughter during this dance lesson warranted further investigation.

In this study of laughter in naturalistic participatory dance contexts, we situate laughter at an interdisciplinary crossroads, asking how it emerges and contributes to behavioral coordination. We use behavioral coordination in a broad sense: not as a single, “unified synchronisation” (von Zimmermann et al., 2018), but as a layered process that spans spatial, temporal, affective, and physiological alignment. Drawing on Glenn's (2003) distinction between “laughing at” and “laughing with,” where the latter typically signals affiliation, playfulness and co-presence, we take affiliative “laughing with” as an important starting point. Laughter in dance appears to be principally self-referential or self-involved, and related to movement as bodies mediate dancers' interactions.

The distinctive—and methodologically challenging—feature of this project is that our dance contexts were not designed around laughter, nor were they recorded in experimental settings controlled for laughter capture. Instead, we analyse observable laughter instances emerging across three different dance situations, each documented for other research purposes:

1. Line dancing lesson: a line dance lesson conducted as part of an academic workshop involving participants with diverse dance backgrounds, and the session that prompted this project.
2. Folk dancing session: an instructed session of Norwegian folk-dancing, a paired social dance form, at a social gathering.
3. Duo Dance sessions: the development and performance of improvisation-based choreographies with two professional contemporary dancers in co-located and networked (remote) settings.

Laughter arose spontaneously within each of these situations. This gives us the opportunity to examine how laughter surfaces and functions in real creative and learning environments, rather than in tightly controlled laboratory settings.

We argue that understanding the cultural and interpersonal value of the arts requires an analytical shift from experimental or stage performance toward the participatory and developmental phases of real-world artistic activity. Laughter, though typically absent from polished performances, may be a crucial element of the creative process. Its role in building interpersonal synchrony and shared sense-making is not merely a by-product of play but a vital support for artistic engagements, including learning, improvising, and creating together. Recognizing this invites a broader and more inclusive understanding of the arts as lived, co-constructed experiences rooted in our evolutionary need to connect.

In this article, we investigate how laughter emerges in these three dance contexts and how it contributes to shared sense-making and behavioral coordination within each group. Using video/movement analysis, physiological measures (heart rate), and participants' phenomenological accounts, we explore these complex behaviors in naturalistic settings. The results of this interdisciplinary analysis offer new insights into how laughter operates at the intersection of movement, affect, and social connection, with functions that are general to many cooperative artistic endeavors and some that are specific to the demands of dance.

### 1.1 Laughter as behavioral coordination in dance context

Theories linking dance and music to social bonding have largely centered on synchrony as a direct mechanism of group cohesion, where moving (together) in time triggers neurohormonal responses that foster affiliation (Tarr et al., 2015; Tarr and Dunbar, 2024). Experimental work supports and extends this logic to other forms of coordinated action: synchronized rowing produces higher endorphin release than training alone (Cohen et al., 2009), walking or singing in step increases cooperation (Wiltermuth and Heath, 2009), and even brief rhythmic tapping in synchrony enhances perceived similarity, compassion and helping (Valdesolo and Desteno, 2011). Within this framework, dance is often treated as a paradigmatic synchronized activity, with music as its principal temporal scaffold.

However, any dance event, particularly in social contexts, is more than bodies entraining to a beat, and the synchrony-cohesion model risks becoming reductive. Studies of dance and interaction increasingly emphasize that coordination unfolds across multiple modalities. For instance, Krug (2022) conceptualizes dance as the coordination of multimodal resources, arguing that synchronization practices operate through complex gestalts of form, intensity, and speed, which cannot be reduced to temporal alignment alone. From this perspective, dancers attune not only to shared timing but also to qualitative dynamics, expressive contours, and kinaesthetic patterns. Tarr et al. (2017) argues that what sets dance apart from other complex, exertive activities is that movement is organized as a direct, intentional response to music, making shared intentionality a crucial higher-order cognitive component. Reddish et al. (2013) show that the combination of synchrony and shared intentionality generates the strongest effects on cooperation and perceived social unity: when people move in time with others toward a common goal, they report stronger bonding and behave more prosocially than when either synchrony or shared goals are present in isolation. These findings highlight that the prosocial effects of dance (and music) depend on high-level cognitive systems–intentions, expectations, shared narratives–alongside low-level bodily alignment (Reddish et al., 2013).

A further challenge to a unitary synchrony model arises from work that shifts attention from strict unison to richer patterns of interpersonal coordination within groups. von Zimmermann et al. (2018) distinguish unitary synchrony, where all members move in lockstep to a common rhythm, from distributed coordination, where patterns of alignment emerge from multiple, overlapping couplings among individuals without an external timing signal. Crucially, their findings show that distributed coordination predicts stronger affiliation and conformity than explicitly instructed unison synchrony, implying that social bonding arises less from perfect temporal alignment than from the fluid, contingent interplay of bodies responding to one another.

Our research builds on these insights by treating dance not as a context that simply produces unison synchrony, but as a multilayered environment for behavioral coordination. Across our three cases, dance events comprised shifting constellations of synchronized and unsynchronised movement, pauses, laughter, talk, negotiation, excitement and occasional tension, all organized around a shared goal. In the duo improvisations, this goal was to sustain interaction and shape a choreography; in the group dances, it was to keep the collective move together just enough for the activity to continue. Following von Zimmermann et al. (2018), we approach coordination as emerging across interconnected dimensions: temporal (timing and rhythm), spatial (proximity, alignment, directional shifts), affective (shared mood, emotional attunement) and physiological (arousal, exertion). In what follows, behavioral coordination refers to this integrated, multi-level phenomenon—including gaps, misalignments and bursts of laughter—rather than synchrony in a narrow temporal sense.

Within this multilayered view, we approach laughter in dance as one mechanism of behavioral coordination among others. Laughter in conversation may produce disruptions through the shared physiological and modal demands of vocalization, whereas in dance, when bodily movement is the focus of interaction, laughter is less often in anatomical conflict, allowing a greater range of laughter intensities to coincide with dance actions without interruption. Instead, it can add a secondary layer of embodied regulation, functioning as an overlay or parallel system nested within the primary kinetic coordination. Moreover, while overlapping speech is often competitive or chaotic, overlapping laughter, even though it varies in duration, acoustic properties, and simultaneity, can occur without being perceived as problematic (Glenn and Holt, 2013). We conceptualize this as layered coordination, a process where the micro-kinetic pulses of laughter are absorbed into, rather than disrupt, the motor flow. This process simultaneously modulates collective arousal and renders emotional states publicly available. Thus, in this second layer, laughter not only supports physiological coupling (through shared breath, exertion, and rhythm) but also facilitates affective coordination, allowing participants to attune to one another's excitement, tension, or relief and to co-create a shared mood, without necessarily being in strict synchrony. One of our aims in this study is therefore to examine how laughter, understood through the lens of layered coordination, shapes group dynamics across three dance contexts.

### 1.2 Evaluation of laughter

Focusing on laughter in dance also raises substantial methodological challenges. Laughter is a pervasive yet ambiguous and complex behavior, and the criteria for describing and evaluating it vary considerably across disciplines. Acoustic approaches emphasize parameters such as frequency, pitch, duration, and tempo (Bachorowski et al., 2001; Krepsz et al., 2024); social and interactional studies focus on context and communicative function (Curran et al., 2018; Rychlowska et al., 2022; Wood and Niedenthal, 2018; Wood, 2020); neurophysiological work differentiates between spontaneous (involuntary, reflex like automatic response) and volitional laughter (more controlled, strategically produced), each associated with distinct neural and endocrine pathways (Belyk and McGettigan, 2022; Bryant et al., 2018; Gervais and Wilson, 2005).

In our study, while acoustic analysis helped us identify instances of laughter, we did not attempt to classify laughter types based solely on acoustic properties or the spontaneous/volitional dichotomy. Instead, we adopted a social functional framework (Wood and Niedenthal, 2018). This approach treats laughter as an ambiguous social signal whose meaning is constructed through situational factors rather than being a direct expression of felt emotion. Its outcome is shaped by social context, verbal information, and other concurrent signals. From this perspective, laughter does not directly represent a specific internal state but, together with other cues, serves communicative functions by influencing listeners' affective states (Curran et al., 2018).

Within this social functional framework, Wood and Niedenthal (2018) argue that representational approaches often fail to account for the diverse elicitors of laughter and its social consequences. Instead, they propose a holistic model that categorizes laughter based on the behavioral intention it conveys and the response it elicits. They identify three primary social functions: reward laughter (reinforcing positive affect), affiliation laughter (maintaining social bonds), and dominance laughter (asserting hierarchy). This framework highlights laughter's adaptive role in managing emotional climates and social dynamics. However, rather than applying these categories rigidly, we recognized that laughter, as an instance of social interaction, is both “context-shaped” and “context-renewing” (Garfinkel, 1967; Glenn and Holt, 2013).

### 1.3 Multimodal communication in context

Existing research on the social functions of laughter convincingly demonstrates the importance of context (Curran et al., 2018; Holt, 2016; Wood, 2018), showing how its meaning shifts and how it participates in interactional processes such as repair, implicature, and irony (Ginzburg et al., 2020), as well as in negotiating social congruity and incongruity (Mazzocconi et al., 2021). Within conversation analysis and interactional linguistics, foundational work by Jefferson (1985) and Holt (2012) has examined laughter as an emergent, sequentially organized phenomenon in naturally occurring interaction. More recent phonetic and multimodal studies have further specified how features of laughter, such as timing, duration, and voice quality, are interactionally managed (Ogden et al., 2026). Following this line of work, we treat features of laughter as affordances: resources for doing things in interaction (Ogden et al., 2026). Building on these perspectives, we approach laughter as a dynamic, multimodal event that integrates physiological patterning, social function, and intentional modulation.

What these interactional studies have not typically addressed, however, is the physiological dimension of laughter as it unfolds in real time. While conversational analyses excel at documenting sequential organization, turn-taking, and embodied conduct, they rarely incorporate biometric measures such as respiration, heart rate, or quantity of motion (Niewiadomski et al., 2013). In the present study, we examine laughter as it naturally occurs in participatory interaction while simultaneously capturing the physiological processes that underpin it.

Group dance contexts offer a particularly rich and demanding interactional framework for studying laughter, as they require participants to coordinate across multiple channels—auditory (music, vocalization), visual (gesture, posture, gaze), and tactile (touch, weight-sharing)—often under temporal constraints imposed by rhythm and movement. As Keevallik (2015) demonstrates in her study of verbal instruction in dance teaching, spoken language is systematically adapted to musical timing and bodily movement, forming a multimodal gestalt that facilitates efficient action demonstration and group coordination. If laughter functions as a communicative resource, we might expect it to exhibit similar adaptations under these multimodal conditions.

**Figure 1:**
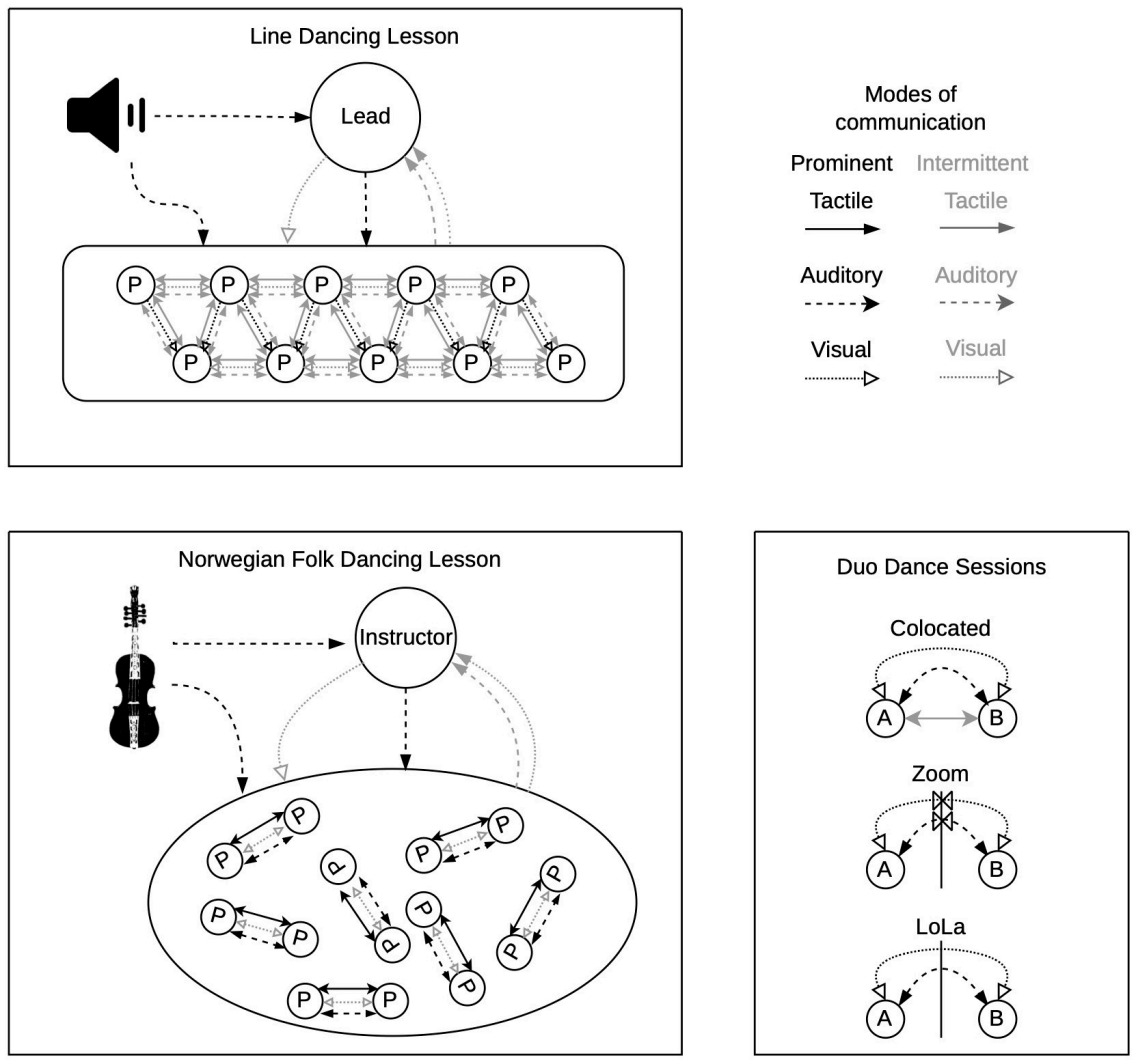
Communication diagrams for the three dance case studies, explaining the directionality, quality, and primacy or intermittency of auditory, visual, and tactile modes of signaling between participants.

Our study examines this possibility across three dance case studies, each selected for its distinctive communicative affordances, specifically, the directionality, quality, and primacy or intermittency of auditory, visual, and tactile modes of signaling between participants (Figure 1). Although a smaller body of work has addressed the multimodal character of laughter, it has largely done so within observer-based paradigms. For instance, Rychlowska et al. (2022) show that participants' accuracy in classifying laughter types decreases significantly when only audio information is available, compared to audio–video conditions. Similarly, Lavan and McGettigan (2017) demonstrate that perceptions of laughter authenticity are enhanced when both auditory and visual cues are present. Together, these findings underline how available communicative channels shape the perception of laughter. However, in these studies, participants remain external observers rather than active participants in the interaction.

In contrast, the dance contexts examined here foreground how different configurations of communicative channels shape interaction from within. By comparing laughter across three conditions, we investigate how its form, timing, and function shift in relation to changing dominant channels of communication, and how laughter circulates and emerges across modalities under these constraints.

Crucially, because our data derive from lived, participatory dance sessions, rather than laboratory tasks or observer judgments, we retain the ecological validity central to the conversation analytic tradition. At the same time, the simultaneous collection of physiological data allows us to examine dimensions of laughter, such as respiratory patterning and autonomic arousal, that are not accessible through audio or video alone. This combination enables us to analyse laughter as it unfolds in real-time interaction, in relation to its communicative goal, temporal placement, relational context, and embodied physiological substrate. Within this framework, laughter bridges affect and action, enabling dancers to align, negotiate, and sustain shared meaning through sound, gesture, touch, and collective rhythm.

## 2 Methods

This study of laughter during dancing activities was not based on project-specific recordings. Instead, it grew out of recordings collected for other projects in which laughter occurred. In each case, the dancers started without a clear idea of how to move together and ended by dancing continuously according to a shared action plan. The prominence of laughing behavior observed across sessions motivated this collaborative evaluation of when, how, and why laughter arose along the way to coordinated movement.

### 2.1 Data collection

Across all case studies, the collaborative dancing activities were captured with audio and video recordings and with continuous physiological measurements. Table 1 describes the quantity and quality of continuous audio–video recordings and physiological measurements per case.

**Table 1:** Recording coverage and duration of coordinating session (performance).

Case study	AV recordings	Durations	Physiology coverage
Line dancing lesson	Audio and partial video	12:30	15 Sensor vests, all
Folk dancing lesson	Audio and intermittent video	13:44	13 Sensor vests, some
Duo Dance sessions			
Co-located	Multi camera video	56:15 (7:28)	2 Sensor vests, all
Zoom	Multi camera video	49:00 (9:20)	2 Sensor vests, all
LoLa	Multi camera video	42:27 (14:20)	2 Sensor vests, all

Consenting participants wore Equivital Life Monitor vests: wearable datalogging sensor vests worn against the skin to measure cardiac activity (two-line ECG, 256 Hz, and RR estimates), respiratory activity (single belt relative chest stretch, 25.6 Hz), and three-dimensional accelerometry from the left side of the wearer's mid-back. Time series of these measurements were aligned between devices and with the audio–video recordings using taps or shake cues embedded in the accelerometry measurements using an established synchronization protocol (Upham and Burnim, 2025).

#### 2.1.1 Physiological measurements

The sensor vest recordings were summarized to facilitate the observation of activity-related coordination. Beat-wise heart rates were normalized (0–1) to match the 10th (0) and 95th (1) percentile values, compensating for differences in cardiac range between participants. The quantity of motion was calculated by downsampling each accelerometry dimension to 10 Hz (after lowpass filtering) and taking the magnitude of the sample-wise difference. These motion features are plotted to show either single participants quantity of motion over time (Duo Dancer 1 and 2, Lead in line dance) or the mean motion across subsets of participants grouped by skill or expertise (self-reported in the Folk Dance session; movement assessed in the Line Dance). As these series often contained high-frequency variation that obscures concurrent measurements, they are sometimes plotted in “cascade,” offset vertically against their own zero line to reduce obfuscation, and with a halo showing group variance (10–90th percentile).

The respiration wave measurements from the Equivital Sensor Vest recordings were also used to interpret participant behavior, including laughter; however, for space constraints, they are not reported here.

In addition to reporting aspects of participants' conditions over time, the physiological measurements are presented as evidence of the movements performed and the coordination achieved with the action plan developed in each session.

### 2.2 Audio analysis

Audio recordings were used to identify the timing of dance events and instances of laughter during each dance session, with and without additional video. Spectrograms of these recordings accompany plots of physiological features to substantiate both the acoustic signatures of specific behaviors by the dancers and the changing acoustic environment across each session. Since music and musical behaviors were important, the constant-Q spectrum was calculated using the Librosa Python library to show log-scale semitone-frequency coverage with a hop size of 44.1 Hz (McFee et al., 2015). In these depictions, laughter bursts appear as broadband noise across the upper frequency range, brighter than concurrent sounds.

### 2.3 Dance activity motion conditions

The dance sessions were segmented by the type of activity being performed moment by moment. Each captured a mixture of communication and motion conditions as participants collaborated to develop a coordinated movement plan. While the differences between cases make it impossible to match the conditions exactly, a few common stages arose:

- Verbal exchange: all sessions included intervals of principally verbal communication between participants, when individuals were mostly still. Verbal intervals in the group dance activities were mainly instructions and directions from the dance instructor to the group, while the Duo dance pair used words to negotiate the structure of their improvised performance plan.
- Gesture introduction: Whether from an instructor physically demonstrating movements (Demo in group cases) or the dance partners suggesting potential material while brainstorming (Search in Duo cases), all dance conditions involved intervals where specific moves or styles were being introduced. This stage always included movement display and observation, sometimes with additional verbal explanations.
- Motion refinement: from the material introduced, the dancers reviewed and refined movement sequences. In groups, they Practiced together each demonstrated move. The duo dancers sometimes Cleaned suggested material and Rehearsed the performance plan.
- Movement sequence run-through: After reaching sufficient understanding and confidence to execute the action plan, participants demonstrated their coordination by dancing to Music in the group conditions and Performing the developed sequence for improvisation.

In addition to documenting the stages of coordination development in each dance session, these event classifications distinguished the modal opportunities for laughter. Verbal exchanges can invite laughter as part of the dialogue and group communication without competing demands for participants' attention. Gesture introduction may provoke laughter of surprise at unexpected moves by the observing participants. Motion refinement requires participants to test their understanding of movement, setting them up to laugh through mistakes, interruptions, and even collisions as they work out how to move with those around them. And while sequence run-throughs have more performance-like pressures, particularly a need to keep dancing despite the occasional disturbances, laughter may arise from the struggle to maintain coordination and satisfaction of success.

Additionally, in the folk dancing session, instances of Applause are also marked for the sake of clarifying the impact of this loud group action on the audio recording.

### 2.4 Laughter analysis

Instances of distinguishable laughter were identified and categorized in all case studies using audio and video analysis with ELAN and Sonic Visualiser (Brugman et al., 2004; Cannam et al., 2010). Instances were first evaluated using contextual and direct evidence regarding the prompts for laughter, the quality of the laughter, and the configuration of participation (order, number), and then recoded according to the identified function of the laughter in these dance sessions.

The group dance case studies primarily used audio recording for evaluation of laughter instances with video references for ambiguous cases, and categorization was at the group level. Duo improvisation case evaluated laughter instances primarily using video and identified individual contributions to them.

The initial categories of evaluation were developed jointly, considering social conditions, communicative goals, and the intensity of laughter behavior. This coding covered:

- Who is laughing, whether an identifiable participant or the estimated proportion of the participants (Solo, Subset, and Group).
- Order of laughter: Simultaneous reactions or contagiously transmitted from one to others.
- Duration of laughter: the number of inhalations in the laughter sequence, approximately.
- Prompt for laughter: Jokes (Verbal and non-verbal), Mistakes, Successes, Uncertainty/confusion, Suggestions/Instructions, Surprises.
- Subject: external object of humor, Self-involved laughter, Mixed participating subject (self and other).

This analysis of instances of laughter then prompted the categorization of laughter by emergent functions in these participatory dance activities. These functions do not fully describe the meaning or impact of each instance of laughter. They are meant to describe their essential contributions in these interactive contexts toward the goal of developing coordination among participants. These functions are:

- Mistake: acknowledge a mistake made, one's own or others.
- Negotiation: express support or resistance in the context of negotiation and group decision making.
- Difficulty: express some degree of trepidation, concern, or confusion about an action being performed or to be taken, without explicitly refusing the task.
- Novelty: acknowledge novelty and surprise at other participants' actions or statements.
- Joyful: express enjoyment of the coordinated movement and success.

The laughter instances were then re-categorized and tagged with their most prominent interpretable function. This evaluation of laughter required two additional categories:

- Other: laughter related to another function or not related to the dance task.
- In Chatter: specific to the group dance case studies, instances of laughter arising in the context of overlapping verbal interactions across the participants, making it difficult to identify a singular function from the recordings available.

The results section reports on the prevalence and distribution of laughter categories and functions in the different dance contexts and presents more detailed interpretations of laughter functions in select excerpts.

## 3 Three case studies

We examine three participatory dance cases, each offering distinct conditions under which participants develop motion coordination. The Line dance lesson follows a group of amateur dancers as they learn a single line dance from basic steps to continuous dancing in unison to the music. The Norwegian folk dance session captures party attendees and participants learning the basic steps of a paired social dance and executing them together to live fiddle music. Finally, the Duo dance sessions track a pair of professional improvisational dancers as they develop a performance plan under different contact conditions, both in the same room (co-located) and across two telematic setups (using Zoom and LoLa, respectively).

These cases differ in social configurations, instructors and groups or professional partners, environmental conditions, limited space to move, visual and auditory interference, and coordination goals, ranging from parallel motion to complementary improvisation. The diagrams in Figure 1 describe these different cases in terms of the modalities of communication available to participants as they develop coordination and dance.

In dance performance, the staging of movements usually prioritizes visual display for the audience, while dancers have to negotiate a changing visual field as they turn and travel. Across these participatory cases, visual signaling and observation were prominent for the improvised Duo Dance sessions, while crowding limited the line of sight for most participants in the lessons. The line dancing participants watched each other to compensate for obstructed views of the lead, while rotations changed who they might see and follow. In the folk dancing session, visual signaling within partners was also constrained by their proximity and relative orientation in different holds, while the independence of pairs made it impractical to directly follow other participants. Visual communication may be prominent at specific stages of coordination development, but it is often disrupted by the actions and environmental conditions of participatory dancing.

The auditory modality was the most reliable channel of communication in the group settings. Verbal instructions from the lead and Instructor could be heard by all participants as they observed or moved, and the groups of participants actively (though intermittently) responded with sounds of their own. In the line dancing lesson, there was very little verbal communication between dancers, whereas the pairs in the folk dancing lesson had to be repeatedly shushed. In the Due Dance sessions, auditory communication was consistently possible (with and without substantial telematic delay) and sometimes quite prominent with one participant singing or both talking through ideas.

Tactile signaling conditions varied the most across cases and sessions. In the Folk dancing, dance pairs touched most of the time, and used the contact to coordinate steps, spins, and changes in holds. In the Line dancing, participants only touched by accident, usually as a result of a mistake. Touch was only possible during the co-located Duo Dance session, where it was incorporated into a subset of the materials performed.

Laughter is a multimodal expression, with distinctive auditory qualities easily broadcast, facial cues accessible to those looking, and bodily vibrations tangible at close range. The social and modal configurations presented in Figure 1 suggest distinct opportunities for this behavior to contribute to the development of coordination, per case, session, and activity.

### 3.1 Line dancing lesson

**Figure 2:**
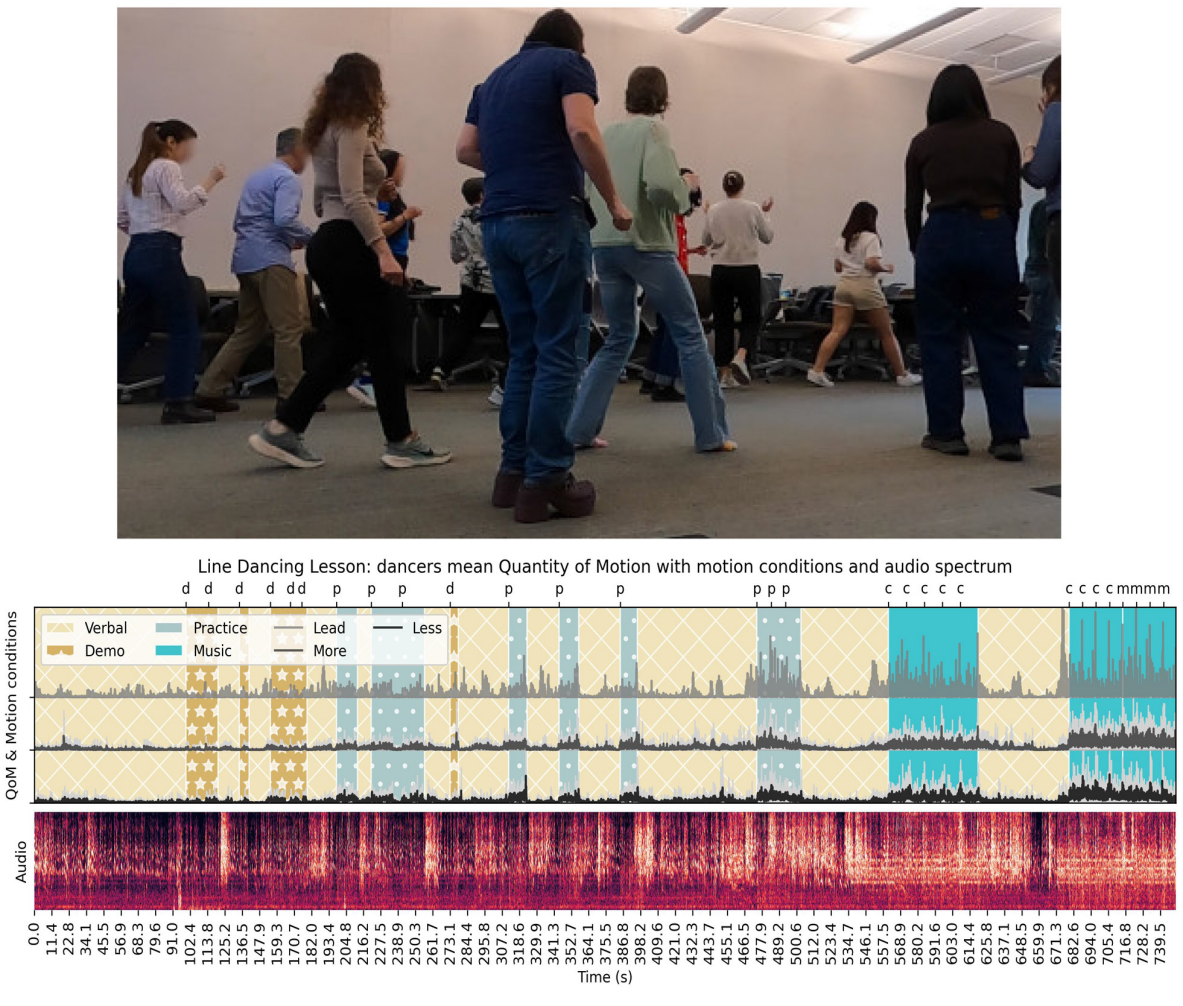
**Above**: Video recording still of dancers during the line dancing lesson, with face blurring. **Below**: Visualization of the line dancing session showing median quantity of motion of each participant group [Lead (1), More coordinated (7), Less coordinated (7), light gray reporting the min and max of QoM] against the sequence of lesson events and motion conditions, with concurrently recorded audio presented with a constant Q spectrogram below.

The line dancing lesson was recorded as part of a workshop on embodied cognition. The lesson was conducted in a classroom adapted for moving together (see the photo in Figure 2). Over 12 min, participants learned a simple line dance from an amateur instructor (Lead), The One Step Forward dance (Wilson and Lucia, N D) to One Step Forward by Band (2014). Participants also contributed to a brief discussion about the experience immediately after the session. In addition to physiological measurements, the event was captured via audio and a single-camera video for the latter half of the session. All 17 participants consented to have their physiological measurements used for research and published anonymously as an open dataset. Data from two participants were excluded from the analysis due to sensor malfunction.

These participants were familiar with each other and the lead, either colleagues or acquaintances of at least a day. None besides the lead had any prior experience in line dancing.

Line dancing requires every participant to move in parallel, performing a short sequence of steps in synchrony with each other (20 beats) against the music (in 4/4), rotating 90 degrees between iterations (four walls). While the sequence being taught was a fixed choreography, the instruction process was improvised and continually negotiated. Spontaneous and solicited feedback from the participants shaped each subsequent learning task, from basic stepping terminology to sequences practiced at increasing speed.

The compound plot in Figure 2 shows the timeline of the session, marking the motion conditions behind the quantity of motion time series. The lesson started with verbal instruction (pale with cross hash), then demonstrations of specific movements (d, brown with star hash) and rounds of practicing the movement sequence with calls from the lead (p). From 560 s, the group danced to the music at a reduced tempo, then at a regular tempo (680 s) with calls from the lead (c), and then continued to dance from memory without calls (m).

A separate analysis of the accelerometer data confirmed that the vast majority of the dancers learned the basic step sequence, synchronizing to the beat, the lead, and each other. To demonstrate the range of coordination success, participants were split into two groups according to their step-wise synchrony with the lead: the More coordinated (7) and the Less coordinated (7). The quantity of motion in both groups grew closer to the lead's levels and structure over successive practice rounds and into the music. The quantity of motion at the end of the session demonstrated that the whole group achieved sufficient coordination to keep dancing through multiple iterations of the taught sequence.

The analysis of these events and the laughter instances was informed by the perspective of both authors, one who acted as the lead at this event, and the other a participant. At the time of recording, neither had begun to consider the relevance of laughter to motion coordination development. The timing of lesson events and instances of laughter was primarily evaluated from audio recordings captured in stereo by a Zoom H6 field recorder. Annotations were collected in Sonic Visualiser. Laughter was identified by audibility, which does not guarantee that all instances were counted. Participation was assessed contextually and with measurements of participants' respiratory activity. When more than one laugher could be heard, the distinction between group and subset was a matter of perceived numerosity and of complementary evidence that some participants were not joining in.

### 3.2 Norwegian folk dancing lesson

The folk dancing session was recorded during an office holiday party. A subset of the dancer participants wore sensor vests during the Norwegian folk-dancing portion of the evening's entertainment. In a 25-min dance session, a professional folk dancer and instructor taught the group component steps of a traditional social dance performed in leader-follow pairs that move in relation to each other through an improvised sequence before separating and finding new partners. Multiple videos and an additional audio recording of the event were captured for research in the public interest. Participants wearing vests consented to data collection and reported their dance training history and experience with Norwegian folk dancing through a short voluntary survey.

Performed to traditional tunes and instruments, here solo Norwegian fiddle, the dance taught was a paired social dance, consisting of an improvised sequence of holds and spins with soft steps to the beat around a crowded dance floor. Participants at this party were colleagues and friends, with two very experienced in this form of dance, a few with passing familiarity, and most new to the style. One of the authors also participated in this dance activity as an initiate. Laughter was not advertised as a central research question to other participants.

**Figure 3:**
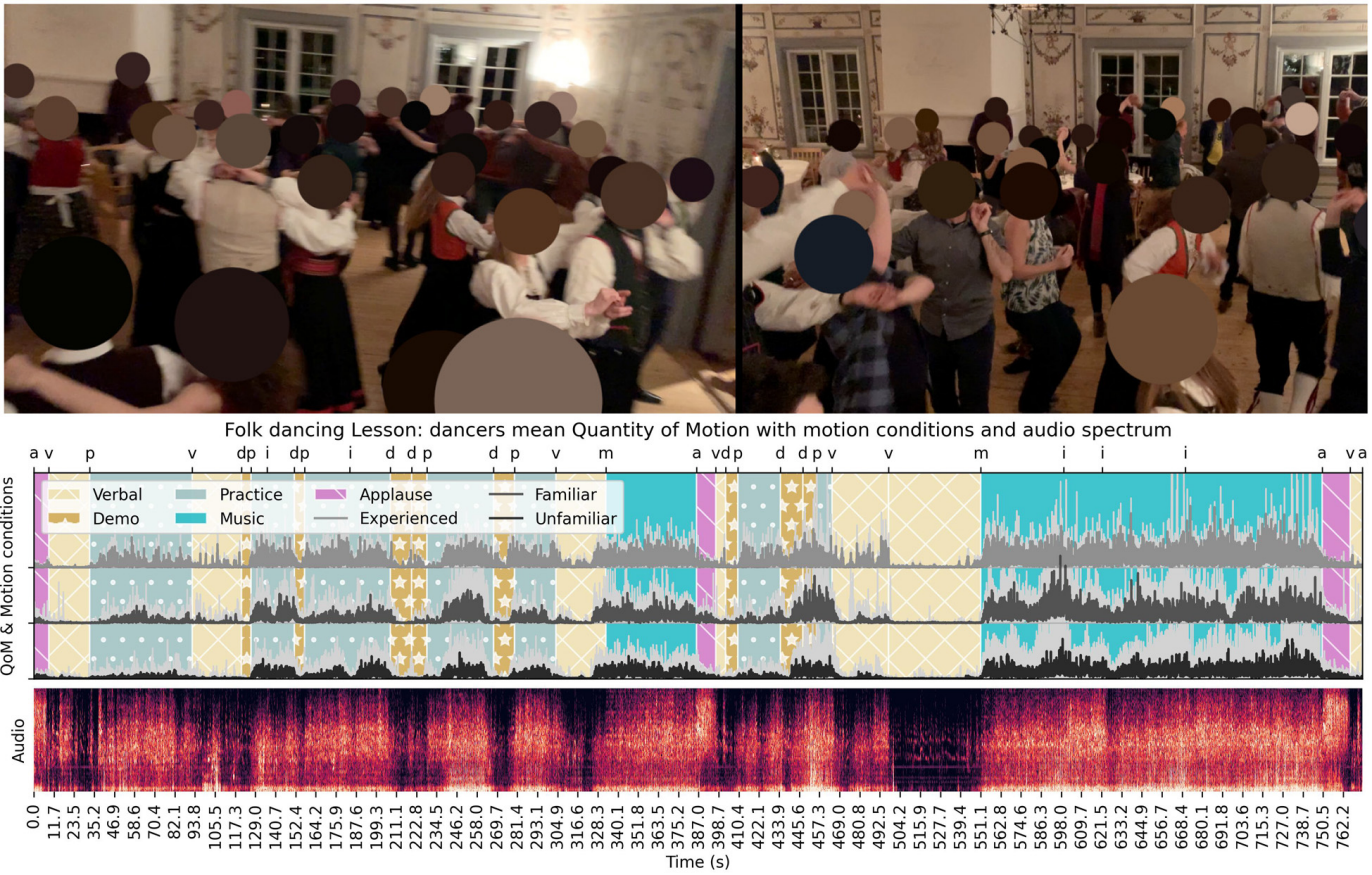
**Above**: Two video stills of the Norwegian folk dancing lesson: while learning an over-the-shoulder hold (left) and while dancing to music with different partners (right) (Photos: Kayla Burnim). **Below**: Visualization of the Folk dancing session showing median quantity of motion of each participant group [Experienced (2), Familiar (4), Unfamiliar (7), light gray reporting the min and max of QoM] against the sequence of lesson events and motion conditions, with concurrently recorded audio presented with a constant Q spectrogram below.

Like the line dancing lesson, the Norwegian folk dancing lesson captured a group of people developing coordinated movement through verbal instruction, demonstrations of specific moves, intervals of practicing together, and dancing to music. Figure 3 shows the progression from the start of the session through the full instruction period and to the cheers after the first full round of dancing with live music (800 s).

Event timing and instances of laughter were primarily evaluated using the audio recording of this dance session (Zoom H6 field recorder), with additional evidence from video captured during the event with an action camera [GoPro (GoPro, Inc., CA, USA)] and from phone recordings. Annotations were added in Sonic Visualiser (Centre for Digital Music, Queen Mary University, UK). Challenging acoustic conditions, caused by dancer pairs chatting, limited the detection and interpretability of laughter instances. Reported instances are of prominent outbursts and exposed reactions, not a complete account; participation was assessed contextually, similar to the line dancing lesson.

### 3.3 Duo Dance sessions

The Duo Dance sessions were captured as part of a study investigating how dynamics of bodily interaction are shaped in telematic environments, using contemporary dancers to test the limits of communication via audio–video streaming systems (Serdar et al., 2026b). Both participants consented to physiological data collection, motion capture, and audio–video recordings for research (Serdar, 2026).

The two dancers were asked to develop a 10-min, improvisation-based choreography under three distinct conditions:

1. Co-located condition: both dancers were in the same studio.
2. Remote Zoom condition: the dancers were situated in two separate studios and connected via Zoom, using large TV screens and room microphones.
3. Remote LoLa condition: the dancers were situated in two separate studios and connected via LoLa, a low-latency, high-fidelity audio-video streaming system, using large projector screens and clip microphones to support more immersive interaction.

**Figure 4:**
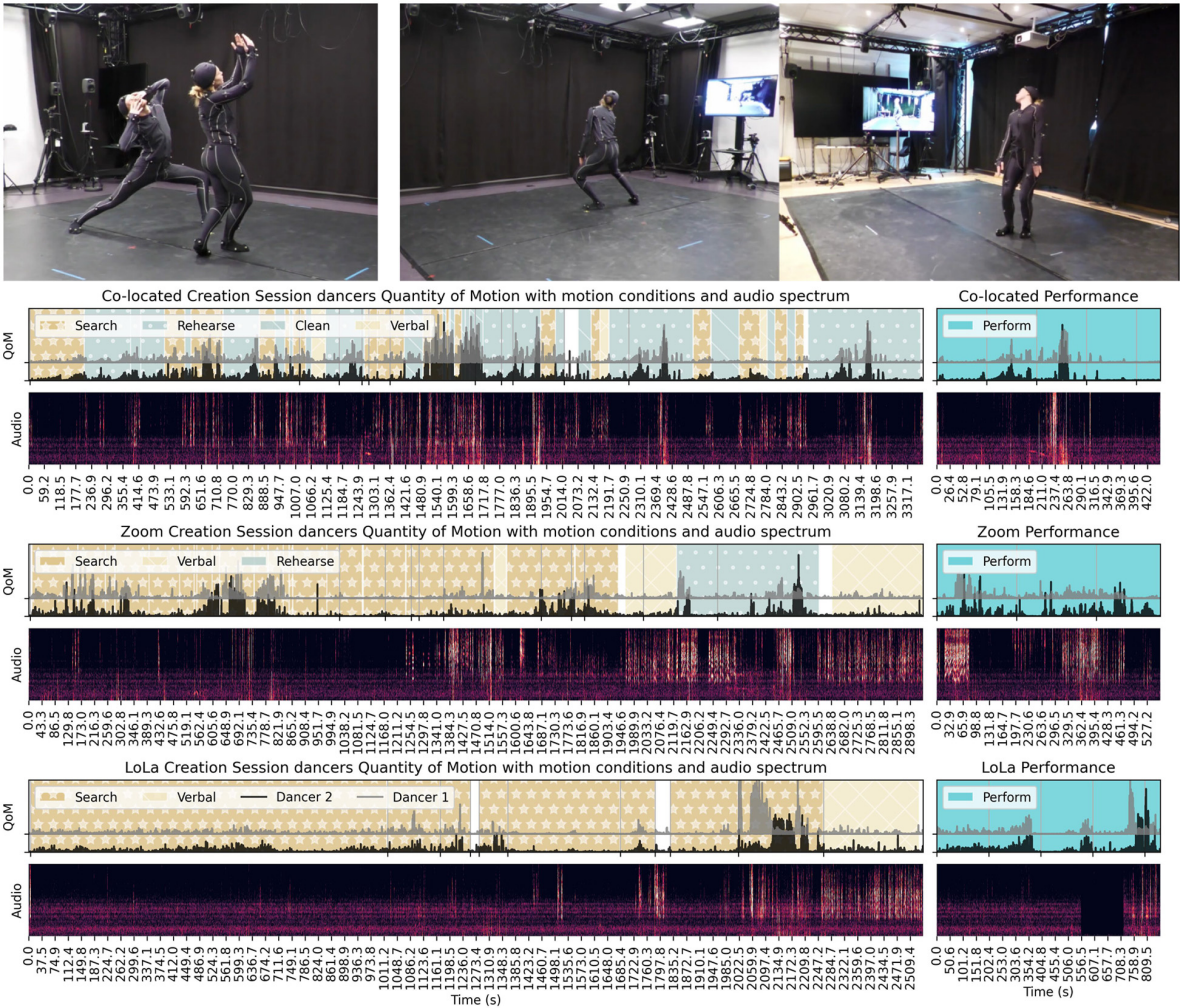
**Above**: Camera views of dancers in the Co-located and the split Zoom conditions for the Duo Dance improvisation sessions. **Below**: Visualization of the Duo dancing sessions showing the quantity of motion of each participant against the sequence of motion conditions structuring each session and concurrent audio below, following the structure of preceding plots. To the left are the measurements taken during the creation of the improvisation performance, as they formulated a plan, and to the right are their movements and the concurrent audio for each performance.

Figure 4 shows video stills from two sessions and compound plots summarizing each session. In all three conditions, dancers were given up to 1 h to develop their choreography (Creation, Left plot) before performing (Performance, Right plot). During the creation phase, dancers were instructed to minimize verbal communication in order to prioritize bodily interaction and kinaesthetic negotiation.

Improvisation-based creation in contemporary dance requires dancers to remain receptive and responsive to one another's proposals, gradually shaping emergent material by returning to, clarifying, and refining shared sequences. This process involves sustained attentiveness, responsiveness, micro-level decision-making, and ongoing negotiation. The study examines how these interaction dynamics differ between co-located and remote settings and explores whether the technical quality of mediation–specifically, the differences in latency and fidelity between Zoom and LoLa–shapes dancers' creative process.

The plots in Figure 4 illustrate how the dancers structured their creative process by visualizing the overall organization of each session's motion conditions alongside the quantity of motion produced by each dancer over time. In the co-located session, the dancers alternated between proposing new ideas (Search) and refining emerging material, with only a few pauses in movement for verbal exchange. In the Zoom condition, most of the rehearsal was devoted to searching both jointly and independently, before the dancers discussed what to perform and tested the material in an extended rehearsal sequence. In the low-latency (Lola) condition, the session was spent almost entirely on searching and exploring movement possibilities, followed by a discussion to establish the final performance structure.

In all cases, the improvised performances were conducted without verbal negotiation or instruction between the dancers. The performances also did not feature any noticeable laughter. Thus, the laughter analysis that follows will report only on the creation sessions.

The structure of creation events (changing motion conditions) and laughter instances was evaluated in ELAN from video recordings (single-view in co-located, dual-view in remote conditions). One of the authors was present for the recording sessions and studied the dancers' interactions according to other criteria before the question of laughter arose. Analysis of a pair of dancers under these controlled conditions enabled more consistent identification of who was laughing and when. In addition to assessing laughter function, instances of laughter in the Duo Dance sessions were characterized as solo (only one dancer), contagious (starting with one dancer and then joined by the other), and simultaneous (both dancers started laughing around the same time).

## 4 Analysis

The following section presents summary figures and tables, along with a focused interpretation of laughter patterns in each case study. Instances of laughter discussed in detail are annotated by letter over the cardiac activity shown in the compound plots.

### 4.1 Line dancing trends

**Figure 5:**
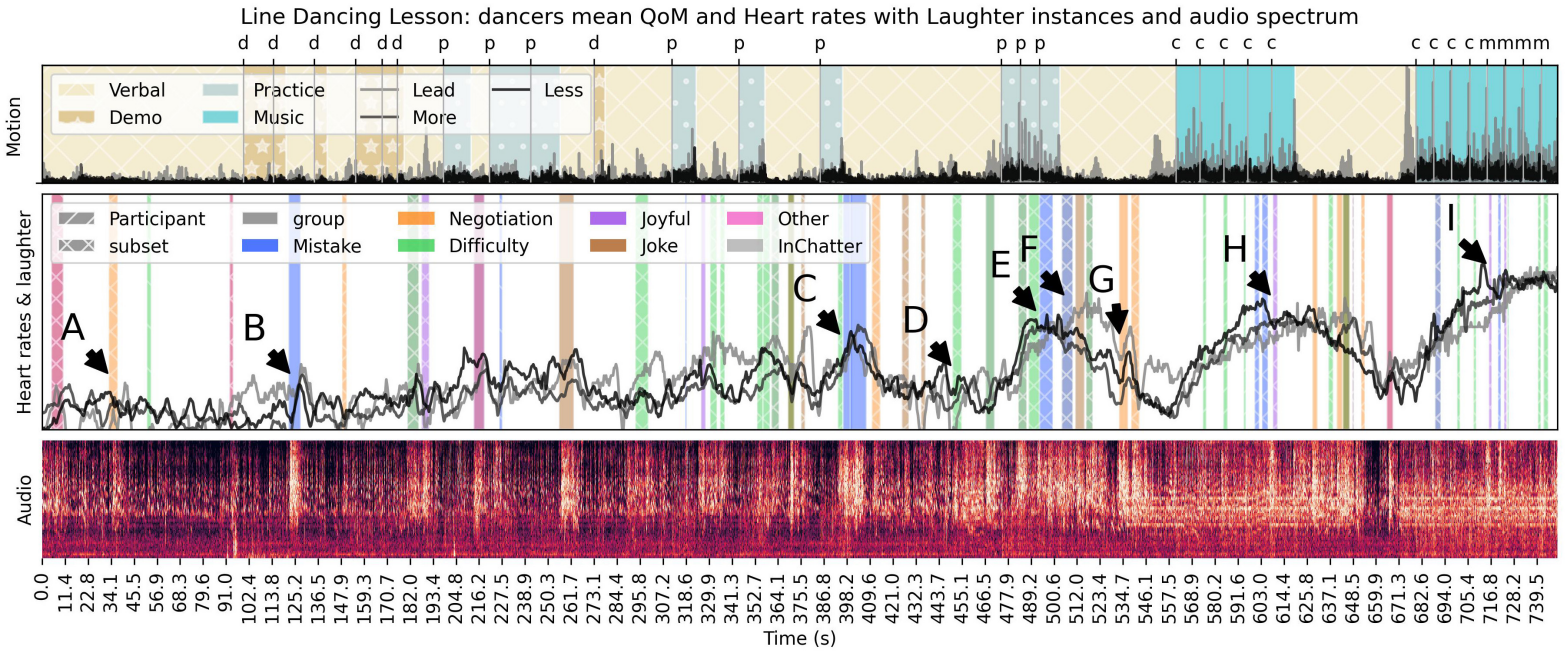
Line dancing lesson sequence showing average quantity of motion **(above)**, average normalized heart rate and laughter intervals **(middle)** and concurrent audio **(bottom)**. Measurements report average across subgroups, Lead (1) and participants showing More coordination (7) or Less coordination (7). Annotations indicating the number of motion measurements mark the lesson events and motion conditions, as described above. Highlighted intervals over heart rate mark instances of laughter by function category (color) and proportion of participants laughing (hash), with lettered annotations described in the text.

A total of 58 instances of laughter were distinguishable in the event recordings, ranging from individual dancers (6) to subsets (41) and the group together (14). Figure 5 highlights these instances in the middle plot, alongside the normalized heart rate measurements for the lead, More coordinated, and Less coordinated participants. The auditory consequences of the laughter can also be seen in the audio spectrogram: bright, wide-spectrum noise when the group joined in.

The categorization of laughter instances was performed by the session instructor, allowing their familiarity with the events to inform the interpretation of how laughter contributed to them. However, when interpreting instances of group laughter, whether as a whole or as a subset, there is still a possibility that multiple distinction functions and prompts influence the behavior of many participants. There are also instances where the context is too ambiguous to retrospectively interpret a cause or function, particularly when the laughter arose “In Chatter,” i.e., while multiple conversations were active at the same time.

Table 2 shows the number of laughter instances per category and motion condition. This group was not experienced in line dancing; however, none of the moves was particularly surprising or difficult, so it is reasonable that there were zero instances of laughter during the demonstrations. Participants could focus on interpreting and copying the slowed-down actions at their own pace. In the practice intervals, when the group danced together to the instructor's calls, laughter mostly arose from aspects of challenge: acknowledging mistakes, signaling feedback on the coordination plan, and showing the difficulty they were facing. When dancing together to the music, these same signals of struggle were prominent with the addition of laughter acknowledging success: giggles mixing with cheers as the group completed the dance sequence and turned to a new wall without crashing. However, across all event types, the most frequent context for laughter was during the verbal exchanges with the instructor.

**Table 2:** Instances of laughter during the line dancing lesson motion conditions by identifiable function.

Condition	Mistake	Negotiation	Difficulty	Novelty	Joyful	Joke	Other	InChatter
Verbal	4	14	14	0	3	5	4	11
Demo	0	0	0	0	0	0	0	0
Practice	3	1	5	0	0	0	0	2
Music	4	0	8	0	3	0	0	1

The category of laughter most expected in the play-like dance learning environment was Mistake acknowledgment. As people try to move in new ways, mistakes and accidental contact are bound to arise. As reported in the first column of Table 2, laughter following mistakes arose during practice and dancing to music. However, mistake-related laughter also carried over from these intervals of motion, as well as arising directly from verbal communication.

The first notable instance of Mistake-related laughter is the burst at 123 s, letter B in Figure 5. The lead was not an experienced caller and occasionally misspoke while giving directions. After incorrectly ending the practice calls with “Step,” one of the participants offered the correction of “Touch” and the laughter outburst from the group followed the lead's verbal acceptance of this correction. The average heart rates of both subsets of dancers showed a larger increase while they laughed through this moment of tension, a faster rise than any dancing-driven change in cardiac activity during the demo and practice intervals. Another moment with Mistake acknowledgment laughter followed a practice round that ended in some participants colliding at 398 s, Letter C in Figure 5. Like the laughter in response to the lead's mistake, participants laughed in reaction to the accident across varying degrees of self-involvement, from those who ended up in physical contact to those who were just catching on to the incident through the response. When mistakes are sufficiently exposed (verbal error) or at a scale to draw the attention of others (collision that interrupts the dance), the group laughter response provokes substantial heart rate changes, a rapid rise and fall.

As described in the diagram in Figure 1, the lead's principal mode of communication was auditory, verbalizing instructions and calls. The lead solicited feedback during the patter of instruction, directly and indirectly asking the group about their comfort with the material being taught or with the plans to continue dancing. Rather than each responding in words, the most common response was laughter, signaling their readiness to participate. These instances of laughter during the verbal instruction served as Negociation: to express agreement or concern about the lead's plans. For example, when the group tittered at the mildly humorous description of their shared goal of “moving in the right direction” (Letter A, 33 s). Regardless of how participants thought of their own behavior in the moment, this audible response from the group signaled to the lead that participants were attentive to direction and open to the challenge ahead.

When group laughter expressed a reaction to information or a plan without solicitation from the lead, it usually indicated Difficulty. Letters D through G in Figure 5 span an interval where the lesson was almost derailed, and laughter from the group helped set it right. Before D, the lead played a bit of the music (Band, 2014) to indicate the dance's target tempo. The laughter at D (450 s) and immediately following (467 s) comes from participants expressing their trepidation about trying to dance the sequences at full speed. While these signals were clear to the lead, it is often necessary during dance instruction to push amateur dancers out of their comfort zones, so the lead began calling the next practice round at 130 BPM (474 s). Most of the group succeeded in completing the sequence twice (two walls); the third round (letter E) fell apart, with mistake-marking laughter spreading as the dancers collided and halted (motion decreased from 495 s). The group broke into energetic chatter and laughter as the practice paused. At 506 s (Letter F), the lead's heart rate increased as they were stressed by the dilemma of how to proceed: repeat the challenge, take things more slowly, review specific moves, or just call it quits. A verbal suggestion to take is slower produced more group laughter out of the chatter (Group Negotiation/Difficulty 511 s, Subset Difficulty 518 s), a sufficient signal of the group's collective willingness to continue the activity. Letter G in Figure 5 points to the group's reactions as the lead teased (533 s) and then played the music at 76 BPM (half speed), a dance condition they firmly rejected with laughter after hearing a bit at 538 s.

When the group managed to dance together to music, participants' laughter was more scattered and fell into two distinguishable categories: difficulty, as dancers struggled to keep up and keep going, and joyful laughter at their success. Joyful laughter at 715 s (letter I) aligned with the group making it through four iterations (all four walls) at the original tempo, and similar laughter emerged at 608 s (letter H) at the slower tempo as dancers successfully returned to their original positions. In both these moments, laughter allowed a subset of participants to mark their sense of accomplishment without interrupting the dance.

Throughout the lesson, most of the laughter was directly related to the dancing tasks, including some more typical Jokes about the experience during verbal intervals. Only a total of four bouts of laughter in 43 interpretable instances (61 total) were not about the activity at hand.

Laughter during the line dancing lesson exposed negotiating functions of laughter, allowing the group to communicate with a lead throughout the challenging task of learning a dance. Laughter together also allowed the group to manage mistakes and share jokes, moments that provoked coordinated increases in heart rate across the group. And laughter was still used by subgroups and individuals during the practice and music-dancing intervals as they navigated the challenge of moving with and around nearby bodies. It seems likely that this lesson would have been much longer and more difficult if the participants hadn't laughed through it.

### 4.2 Folk dancing trends

The Norwegian folk dance lesson shares many of the same conditions for laughter as described in the Line Dancing lesson; however differences in the dance configuration and the lesson environment (as shown in Figure 1) altered the balance of laughter composition and function.

This participatory dance session included substantially more overlapping inter-dancer communication, with laughter popping out of the sound mass of pairs trying to learn, coordinate, and socialize all at once. Of the 59 instances of laughter identified, 13 were solo laughter, 41 were subsets, and only five involved the group as a whole. The “In Chatter” category of laughter is the most frequent, reported in Table 3, with a subset still having discernible functions.

**Table 3:** Instances of laughter during Norwegian folk dancing lesson motion conditions by identifiable function.

Condition	Mistake	Negotiation	Difficulty	Novelty	Joyful	Joke	Other	InChatter
Verbal	0	2	1	2	1	4	0	4
Demo	0	1	2	1	0	0	1	2
Practice	1	0	5	0	0	0	0	17
Music	0	0	1	0	0	0	0	22

**Figure 6:**
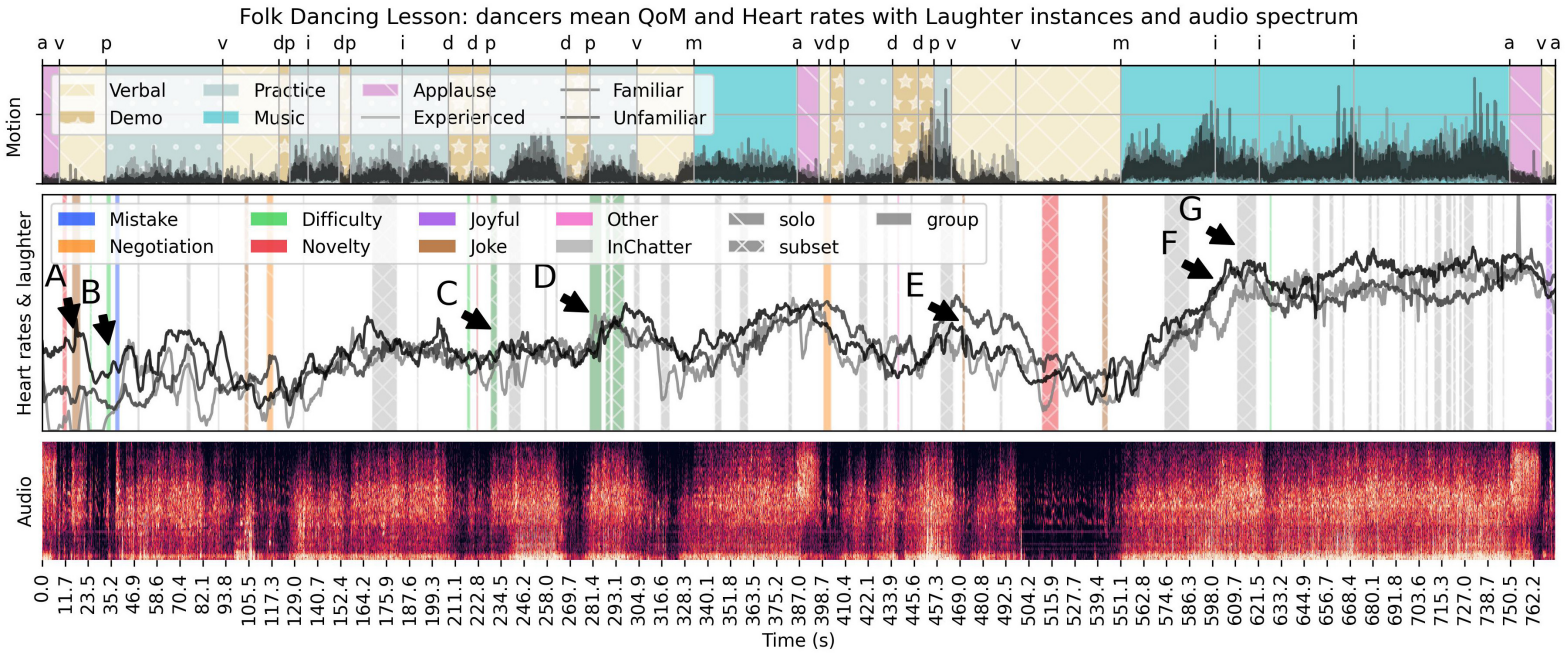
Norwegian folk dancing sequence showing average quantity of motion **(above)**, average normalized heart rate and laughter intervals **(middle)** and concurrent audio **(bottom)**. Measurements report averages across subgroups: Experienced (2), Familiar (4) and Unfamiliar with this dance style (7). Annotations behind the quantity of motion measurements mark the lesson events and motion conditions. Highlighted intervals over heart rate mark instances of laughter by function category (color) and proportion of participants laughing (hash), with lettered annotations described in the text.

Figure 6 shows the average heart rates of each participant subgroup (Experience, Familiar, and Unfamiliar with this style of dance) as well as the laughter instance identified in the recordings. The average normalized heart rates for all three subsets of dancers are remarkably similar throughout the session, rising during stretches of continuous dancing and falling when they listened to the instructor.

Laughter started early in this dance lesson. Letter in A in Figure 6 points to a burst at 16 s that followed the instructor stating “I hear you are all up for a little dancing lesson,” with typical Norwegian understatement. Laughter across the group at this mild joke also served as group Negotiation, demonstrating to the instructor that he had the group's attention and signaling some of their trepidation. At Letter B (32 s) the group laughed at the prospect of finding a dance partner, a social challenge, and then laughed more at 36 s as they realized the instructor's expected them to find one now (Mistake). The increase in heart rate that followed may have been as much about the social stress of selecting a dance partner in a mixed-work setting as it was about their locomotion around the dance floor.

Learning to move in pairs was more difficult for some moves than for others, and this was reflected in the intensity and frequency of laughter heard across the group as they practiced. Letter C of Figure 6 points to a subset laughing they attempted at an over-the-shoulder hold (223 s) and Letter D to a much larger group laughter responses to the more difficult Pancake spin (280 s). The Pancake involves partners holding both hands while each takes a full turn under these two points of contact, and participants laughed through the awkwardness of bumping into each other and getting stuck in weird poses as they figured it out. While the group's attention was scattered in this paired practice condition, consternation was a discernible tenor in the laughter heard across the crowd. Thus, these laughter bouts were labeled “Difficulty” and “In Chatter” to allow room for mistakes, negotiation, novelty, and even joy to also contribute as they navigated their entanglement.

In paired social dancing, the coordination task is not only a matter of conforming to a dance style by executing established moves to the music. There is also a need to maintain coordination with your dance partner, and every new partner is a new challenge. Every time the instructor mentioned the task of finding partners, the group responded with laughter (A 32s, B 36s, E 469s in Figure 6), marking this necessary part of Norwegian Folk Dancing as a common reason for trepidation. The last instance of this task arose at Letter F (no laughter) in Figure 6, when participants were directed by the instructor to change partners in the middle of dancing to the music. The command at 599 s prompted an immediate reduction in movement, while the heart rates of the less-experienced dancers continued to rise. As the dancers managed to recouple, chatter on the dance floor ballooned (Audio, 604 s), with laughter cutting through the sound mass (Letter G, 610 s) as they struggled to coordinate again. The need to communicate with new partners was so strong that the babble overwhelmed the fiddler in the corner, and the group had to be shushed (622 s) before the music could be restarted. While the volume of talking was diminished from then on, laughter was audible across the crowd for the remainder of the dance.

Two additional categories of group laughter arose while the dancers were not preoccupied with moving in pairs. At 504 s in Figure 6, the instructor prepared the group for a full round of dancing to the music by taking a moment to describe the traditional conditions of this dance form. The pre-modern setting he described prompted Novelty laughter from a subset of participants. And at the end of this dance, at 7, Joyful laughter emerged after more direct cheers, indicating enjoyment by at least a subset of participants.

The folk dancing session, with substantial but manageable environmental constraints on mobility, visibility, and audibility, featured laughter from beginning to end. During verbal and demonstration intervals, dancers expressed collective attention and trepidation, acknowledged a mistake, and expressed joy through laughter. During practice and music intervals, they also laughed frequently during full-body motion and verbal communication as they moved in pairs. The overall level of laughter across the group seemed to correspond to the difficulty of specific moves and the challenges of coordinating with new dance partners.

### 4.3 Duo Dance trends

**Figure 7:**
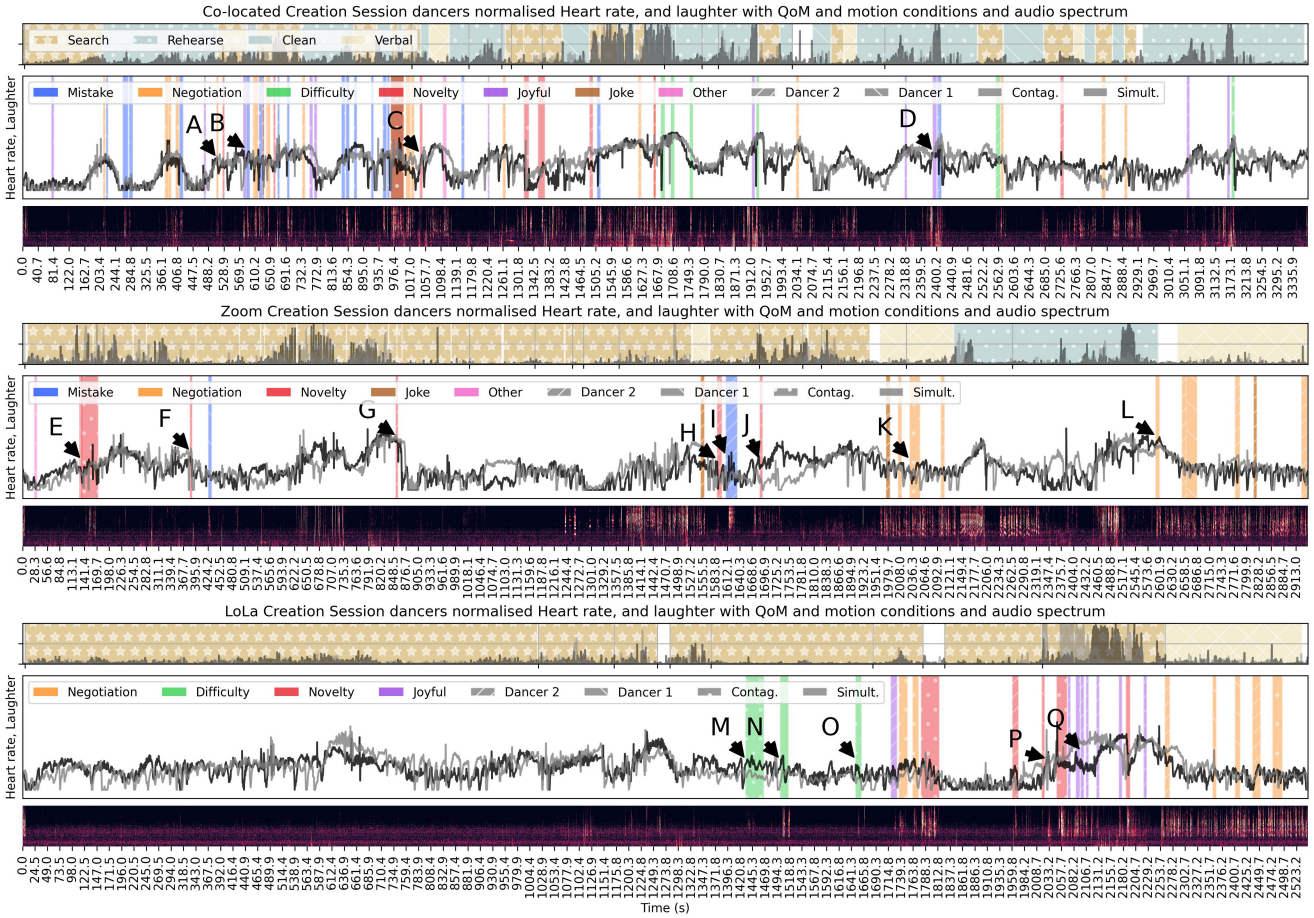
Summaries of the three Duo Dance improvisation sessions, creation section only, showing quantity of motion, normalized heart rate and laughter intervals and concurrent audio for each creation session. Annotations behind the quantity of motion measurements mark the creation events and motion conditions. Highlighted intervals over heart rate mark instances of laughter by function category (color) and participants (hash), with lettered annotations described in the text.

The environmental context (see diagram in Figure 1), recording conditions (multiple cameras, clear dance floor), and social simplicity (only two dancers) facilitated a more detailed study of laughter in the Duo Dancing sessions than the group dancing lessons. The timing of dance activities and laughter in all three Creation sessions is presented in Figure 7.

#### 4.3.1 Laughter participation counts

When we compare laughter across the three conditions, the most striking differences we observed were in how often dancers laughed and whether the laughter was shared (i.e., temporally overlapping, identifiable laughing behavior). Table 4 shows the number of laughter events coded as solo, shared-contagious, and shared-simultaneous in the co-located, Zoom, and LoLa conditions. While shared-contagious implies that laughter began with one dancer and spread to the other, shared-simultaneous implies that they began laughing at the same time. This table highlights how the emergence and circulation of laughter are affected by available modalities and communication channels.

**Table 4:** Laughter participation counts across Duo Dance recording conditions.

Participation	Creation phase			Performance
	Colocated	Zoom	LoLa	All sessions
Solo	28	16	12	0
Shared, contagious	20	4	10	0
Shared, simultaneous	16	0	1	0

In the co-located condition, shown in the top set of plots in Figure 7, laughter was highly interactive: of the 64 laughter instances, more than half involved shared forms as 20 contagious and 16 simultaneous laughs, alongside 28 solo laughs (Table 4). While five of 28 solo instances were short chuckle-like laughter while moving, contagious laughter arose when one dancer's laughter triggered the other's (20), creating a feedback loop of mutual playfulness and responsiveness. Simultaneous laughter emerged when both dancers recognized a humorous moment at the same time–such as bumping into each other (letter A in Figure 7, 508 s), making mistakes (Letter B, 585 s), or responding to unexpected suggestions (letter C, 1042 s). Because these moments were jointly perceived, laughter functioned as a marker of temporal alignment and embodied co-presence. In this setting, the full spectrum of multimodal communication, including eye contact, subtle vocal tones, breathing patterns, micro-movements, and physical contact, remained intact. This allowed laughter to circulate fluidly across visual, auditory, and kinaesthetic channels.

In contrast, these multimodal cues were fragmented in the remote conditions due to physical separation and the limitations of technological mediation. In the experiment, LoLa's low-latency, high-quality audio and video transmission, together with the use of larger screens, potentially supported better temporal and spatial coordination. In the Zoom-mediated condition, the researchers intentionally used a smaller TV screen rather than a large projector in order to create a more restricted interactional environment. This design choice allowed us to systematically examine how a reduced visual scale, together with higher latency and lower audio and video fidelity, shapes the affordances of the technical setup and, in turn, impacts the availability and circulation of multimodal cues.

In the Zoom-mediated improvisation, the fragmentation of multimodal cues was most pronounced. Laughter was dominated by solo instances (16), while shared laughter dropped to four events, and no simultaneous laughter occurred (Table 4). Two of the shared-contagious laughter events were coded as novelty-related laughter (letter E around 133 s and letter F around 382 s), emerging when the dancers reacted to one another's movement suggestions. The remaining two were coded as negotiation-related laughter (Letter K around 2028 s and letter L around 2591 s), occurring when the dancers attempted to reach an agreement on how to proceed.

Here, higher latency, reduced audio–video synchrony, and the spatial split between viewing and being viewed combined to constrain mutual sensing. The dancers frequently struggled to locate the other's attention or affective response, as gestures and laughter no longer coalesced into a shared perceptual frame. As a result, solo laughter became more pronounced, and it often took on a more introspective, self-directed character, remaining unnoticed by the remote partner unless amplified by verbal cues or larger bodily movements.

In the LoLa-mediated rehearsals, laughter remained frequent but became somewhat less synchronized: out of 23 laughter events, 12 were solo, 10 were contagious, and only one was simultaneous (Table 4). Thus, although LoLa's low-latency, high-quality audio–video transmission still supported shared laughter, there was a noticeable shift away from tightly aligned simultaneous episodes.

The simultaneous laughter instance started at (letters M, N, O), and was coded as difficulty-related laughter (Figure 7, lower set). This moment emerged after extended periods during which the dancers worked silently to generate movement material, primarily taking cues from one another through the screen while facing or looking toward the displays. The laughter occurred when both dancers were attempting to maintain visual alignment through the screen, but their bodily positions became increasingly complex. Rather than interrupting the interaction, the laughter functioned as a shared acknowledgment of the difficulty of coordinating these embodied actions through mediation.

We interpret the decrease in simultaneous laughter in the LoLa condition as an effect of subtle distortions in micro-temporal cues, such as gaze shifts, breath timing, and fine-grained movement synchronization, as well as the spatial disjunction between where the dancers looked (toward the screen) and where they were visually captured by the camera. This introduced a mild misalignment between perception and projection, slightly destabilizing the immediacy of embodied reciprocity. At the same time, both remote settings (Zoom and LoLa) eliminated tactile interaction, including accidental touches and bodily collisions, which in the co-located and group dance conditions frequently prompted simultaneous laughter. The absence of these shared tactile disruptions further reduced opportunities for laughter to emerge immediately and mutually across performers.

The comparison across the three conditions indicates that the emergence and circulation of laughter depend not only on the interactional context but also on the mode of communication that mediates it. Even within a similar improvisational task, differences in latency, audio–video quality, screen scale, and the symmetry of perceptual access shaped whether laughter remained individual, became contagious, or emerged simultaneously as shared affect. While both remote conditions constrained the circulation of laughter compared to co-located interaction, the LoLa setting afforded shared laughter more readily than Zoom, supporting more moments of contagious and simultaneous laughter through its lower latency, higher audio–video fidelity, and more stable sense of temporal reciprocity.

Notably, no laughter occurred during the final performances in any of the three conditions. This suggests that laughter functioned as a rehearsal-specific phenomenon within an embodied mechanism of exploration, negotiation, and attunement, rather than as a performative gesture intended for an audience. Once the structure and relational rhythm of the piece had been established, the dancers appeared to channel their playful reciprocity into movement itself, allowing laughter's affective traces to persist as embodied memory rather than explicit expression.

#### 4.3.2 Functions of laughter

The identifiable functions of laughter also varied with the motion conditions in these sessions, as shown in Table 5. While not every function appeared in every condition, the distributions reflect how different communicative affordances across the three contexts shaped the emotional and interactional landscape of the creation phases for these improvisations.

**Table 5:** Functions of laughter per motion condition in each Duo Dance rehearsal session.

Session	Event	Mistake	Negotiation	Difficulty	Novelty	Joyful	Joke	Other
Colocated	Search	7	9	1	8	3	1	0
	Rehearse	9	7	3	0	9	0	0
	Clean	3	3	2	3	0	1	0
	Verbal	0	0	0	0	0	0	1
Zoom	Search	2	0	0	7	0	0	1
	Rehearse	0	1	0	0	0	0	0
	Verbal	0	10	0	0	0	3	0
LoLa	Search	0	2	3	5	8	0	0
	Verbal	0	5	0	0	0	0	0

In the co-located condition, laughter was both frequent and functionally diverse. It occurred most often in relation to negotiation, novelty, and mistakes, with additional instances linked to joyful moments (Table 5). Many laughs followed moments of imbalance, mis-timing, or unexpected contact, which were quickly reframed as shared amusement through eye contact, touch, or brief gestural exchanges. We coded negotiation when dancers accepted or rejected each other's suggestions, or when they had not yet settled on material and were on the verge of proposing what should come next. In these moments, laughter typically softened disagreement or signaled tacit acceptance of a proposal, allowing decisions to be made without explicit verbal explanation. Occasional jokes and “other” idiosyncratic triggers appeared only rarely, primarily during search phases and brief verbal exchanges. Although the dancers kept verbal communication to a minimum, short utterances such as “OK!” or “nice!” often accompanied laughter, commenting on the situation rather than directly causing it.

As shown in Figure 7, during the co-located session, the dancers moved repeatedly between searching for material, cleaning it, and rehearsing it. Two dancers' heart rate and motion peaks largely aligned with these phases. Laughter occurred in all activity segments but clearly decreased toward the end of the rehearsal. This pattern suggests that laughter was most prominent during phases of emergence, when dancers were dealing with unfamiliar material, responding to each other's proposals, and negotiating what to keep. As the choreography became more stable and rehearsed, both the number and frequency of instances of laughter diminished.

In the top set of Figure 7, the segment between 2,389 and 2,430 s (letter D), corresponds to a rehearsal moment in which the dancers perform a tightly synchronized house-style sequence combining footwork, hand clapping, and a turn. This coordinated activity is mirrored in the quantity of motion plotted above, where aligned peaks signal high degree of temporal coupling between the performers. Although this section appears demanding in terms of coordinating steps and claps, we still observe joyful laughter (2,389 s) and laughter related to mistakes (2,430 s) during this segment. This moment exemplifies how laughter can emerge as an additional layer of coordination without disrupting the shared flow of movement. Instead of signaling frustration, these laughs mark willingness to continue, enjoyment of the challenge, and mutual commitment to the task.

In the Zoom-mediated condition, the rare laughter was more tightly linked to verbal interaction (Table 5). Most laughter occurred during verbal negotiation exchanges (10 instances), while other laughter arose during moments of novelty in the search phase or in response to jokes, with only a few instances related to mistakes. Notably, no laughter was coded as difficulty- or joy-related in this condition. Many episodes appeared to arise from communication problems or attempts to interpret the partner's intentions through the delayed, visually constrained video stream. The motion subplots of Figure 7 further shows that, similar to the LoLa session, the Zoom creation session was dominated by search activity until the final quarter, when a brief period of verbal negotiation followed by a short rehearsal. Heart-rate and motion peaks were less tightly aligned than in the co-located condition, and laughter clustered around verbal segments rather than being distributed across movement phases.

To illustrate, the segment around 1,608 s (letter I) captures a moment in which Dancer 2 laughed after accidentally approaching the recording camera, rather than the Zoom camera, while attempting to interact with the other dancer. Dancer 2, together with those in the local room, including the researchers, burst into laughter, whereas the remote partner (Dancer 1) remained unaware of the incident. In this middle plot, the blue area marks this mistake-triggered laughter: without additional motion, the heart rate of Dancer 2 increased with the onset of laughter, whereas the heart rate of Dancer 1 in the other studio remained unchanged, and their movement continued unaffected.

In the same plot, around 1,685 s (letter J), Dancer 2 performed larger fencing-like movements and chuckled in response to Dancer 1's small facial movements close to the camera. Similar to the previous example, this novelty-prompted laughter fails to circulate to the other studio. Playful gestures also occasionally triggered laughter that remained local and did not travel across screens, such as at 853 s (letter G) and 1,587 s (letter H) in Figure 7. By contrast, verbal jokes or ironic comments were among the few actions that reliably elicited reciprocal laughter from the remote partner (2,028 s letter K and 2,591 s, letter L). In this Zoom setting, laughter functioned less as shared bodily resonance and more as a form of self-regulation, used to express and release frustration or tension while also serving as a negotiation tool to maintain interpersonal rapport despite remote conditions.

In the LoLa-mediated condition, laughter occurred mainly in the search phase and was strongly associated with novelty, joy, and negotiation (Table 5). The dancers often engaged in playful, game-like exchanges, such as miming objects, exaggerated spatial propositions, or jump-rope-like movements, where laughter signaled imaginative engagement and mutual enjoyment, rather than repairing breakdowns, as was more common in the Zoom session. Difficulty-related laughter arose when motor coordination or spatial orientation became challenging (letters M 1,432 s, N 1,500 s, and O 1,650 s in bottom plots of Figure 7), but these episodes were usually absorbed into the ongoing play rather than interrupting it.

As shown in the lower plots of Figure 7, the dancers spent a substantial portion of the LoLa rehearsal engaged in extended improvisational search, followed by verbal decision-making to establish the choreography. One striking feature is that laughter did not emerge until the second half of the creation process. The relatively high counts of joyful and novelty-related laughter during this later phase suggest that LoLa's low latency and high audio–video quality supported pleasurable co-discovery, even as decisions about fixed material increasingly shifted from intuitive bodily exchange toward verbal negotiation.

The moments illustrated in Figure 7 around 2,048 s (labeled as novelty, letter P) and 2,096 s (labeled as joyful, letter Q) provide examples from the search phase of the dancers' creative process, demonstrating how high-fidelity technological mediation through LoLa could still afford shared attunement at a distance, and how laughter contributed to this process. Around 2,048 s, one dancer proposed an imaginary rope and began swinging it, while the other immediately accepted the suggestion and started “jumping” the rope. In this exchange, laughter served as an intuitive mechanism of acceptance, shared by both partners. The dancers continued the playful interaction for some time, alternating roles between jumper and rope-swinger, while the accompanying laughter around 2,096 s signaled mutual enjoyment and helped establish a shared playful atmosphere despite the lack of precise motor alignment.

The motion plot in Figure 7 further shows that, although their movements were not synchronized during these role exchanges, laughter still operated as a coordination mechanism. It enabled the dancers to meet temporally through shared pulses of laughter and affectively through a sustained sense of attunement and co-presence, despite the absence of precise motor synchrony.

Taken together, these findings indicate that the capacity of laughter to circulate between performers—transforming from individual amusement into shared affect—depends not only on latency and technical fidelity but also on the coherence of the perceptual space that supports embodied reciprocity. The co-located condition provided a fully shared perceptual field in which visual, auditory, kinaesthetic, and spatial cues could continuously reinforce one another. This enabled laughter to circulate fluidly between performers and supported the emergence of a shared affective environment. Both remote settings, by contrast, only partially supported such multimodal integration. The LoLa-mediated condition, with its higher technical fidelity, lower latency, larger screens, and more immersive setup, still afforded partial access to shared perceptual cues and supported moments of mutual affective attunement, although less seamlessly than in the co-located setting. The Zoom condition, however, with its higher latency, lower audio–video fidelity, and less immersive mediation, accentuated perceptual fragmentation more strongly. This weakened the sensory feedback loops through which dancers tracked one another's attention, timing, and affective states, thereby constraining the emergence of shared laughter and “performative togetherness” (Serdar et al., 2026a).

## 5 Discussion

This study investigated emergent laughter behavior across three distinct dance contexts using multimodal recordings and close analyses of interaction conditions. By integrating audio, video, physiological measurements, and situated participant perspectives, we examined laughter not as an isolated emotional reaction but as a dynamic interactional resource embedded within collaborative movement practices. This multimodal strategy responds to existing research highlighting the limitations of categorizing laughter through restricted communicative channels alone (Rychlowska et al., 2022; Lavan and McGettigan, 2017), as well as broader calls within laughter studies for richer ecological and multimodal datasets (Niewiadomski et al., 2013). We first took a descriptive perspective to understand laughter in group dynamics, considering identity, order, duration, prompt, and social referent. Our findings suggest that laughter functions as a mechanism of layered coordination, supporting dancers as they negotiate uncertainty, regulate affect, maintain affiliation, and sustain interaction while developing coordinated movement together.

The analyses across the three case studies demonstrate that laughter in dance is not simply a by-product of enjoyment or play. Rather, laughter itself becomes part of the coordination architecture of participatory dance activity. Our findings suggest that, within dance contexts, the modal organization of interaction often allows laughter to coexist with ongoing movement and coordination in distinctive ways. Dancers frequently laughed while continuing to move, improvise, negotiate, and adapt, rather than suspending the activity altogether. In this sense, laughter acted as an additional layer of embodied regulation nested within the broader coordination process.

Across all cases, laughter was rarely disaffiliative or mocking; instead, it played an integral role in sustaining coordination. It repeatedly emerged at moments of uncertainty, challenge, or transition: when dancers negotiated decisions, acknowledged mistakes, responded to novelty, managed social awkwardness, or recognized successful coordination. Rather than merely reacting to humor, participants frequently used laughter interactionally to regulate collective activity while continuing to move together. In the duo improvisations, laughter softened disagreement, signaled tacit acceptance or resistance toward movement proposals, and helped dancers continue exploration without always requiring explicit verbal clarification. In the group dance lessons, bursts of collective laughter conveyed shared trepidation, a willingness to continue, and a mutual recognition of difficulty. These laughter episodes also shaped the pacing of instruction and reinforced participants' commitment to ongoing participation despite mistakes, hesitation, or failed coordination. Across the cases, laughter therefore became embedded within the practical work of developing coordination together, contributing to the negotiation, maintenance, and repair of collaborative movement activity.

This finding extends interactional studies of laughter (Jefferson, 1985; Glenn, 2003; Holt, 2012) into dance contexts, where multimodal coordination depends on the continuous organization of movement, attention, timing, and bodily responsiveness. Previous research on dance interaction has similarly shown that coordination emerges through multimodal and dynamically negotiated processes. Krug (2022), for example, demonstrates how dancers continuously organize interaction through temporal procedures such as delays, accelerations, and accentuations, while Keevallik (2015) describes grammar in verbal instructions itself as a “coordinative device” that cuts across modalities and enables participants to navigate complex physical and social interaction in real time. Our findings extend these perspectives by suggesting that laughter functions in a comparable way: not outside multimodal coordination processes, but as part of them. Just as language and temporal adjustments help organize collaborative activity, laughter contributed affective, physiological, and interactional layers to the ongoing coordination of movement and shared sense-making.

Importantly, the findings also challenge narrow synchrony-based understandings of social bonding in dance. Although synchronized movement undoubtedly played a role in these activities, the coordination observed across the cases was rarely reducible to strict temporal unison. Instead, dancers continually shifted between alignment and misalignment, movement and hesitation, exploration and repair. Laughter frequently emerged precisely within these unstable moments. This supports perspectives that conceptualize dance coordination as distributed, multimodal, and dynamically negotiated rather than as simple rhythmic entrainment (von Zimmermann et al., 2018; Krug, 2022). In this context, laughter became one mechanism through which participants maintained affective and interpersonal attunement despite imperfect synchrony. Our findings, therefore, align with research that expands the social effects of dance beyond temporal alignment alone to include shared intentionality, adaptive coordination, and collaborative responsiveness (Reddish et al., 2013; Tarr et al., 2017).

The diagrams of Figure 1 highlight how the three dance contexts differed in their communicative channels and interactional organization. These varied conditions allowed us to examine not only the role of laughter in coordination, but also how its emergence and circulation were shaped by different communicative ecologies. In the duo dance cases, we compared co-located interaction, in which dancers shared auditory, visual, tactile, and spatial access, with remote conditions, in which communication was reduced to digitized audio-video streams. This comparison allowed us to investigate how the richness and reciprocity of communicative channels shaped laughter beyond its mere perception. In the group dance activities, all of which were situated in shared physical spaces, we further observed how different interactional contexts (e.g., instructional vs. couple-based dance settings) influenced the flow and direction of communicative cues, including laughter.

Dancing in physical proximity, in the co-located Duo dance session and in the group dance activities (Line Dance and Norwegian Folk Dance), allowed dancers to share a common perceptual environment in which visual, auditory, spatial, tactile, and kinaesthetic cues continuously reinforced one another. Laughter circulated fluidly across these overlapping modalities and frequently emerged even as dancers were already engaged in demanding movement. Efforts to coordinate movement together often generated moments of hesitation, incorrect recall, scale mismatches, timing disruptions, or disorientation. Such deviations threatened the continuity of coordinated action. Participants frequently responded through laughter without suspending the activity; laughter also accompanied moments when the movements had to stop (crashing together, getting entangled in paired dancing). During simultaneous movement, laughter often functioned to signal mistakes, acknowledge difficulty, share enjoyment, or soften disruption without requiring the cognitive and attentional demands of explicit verbal negotiation. In this sense, laughter operated as a low-demand communicative resource embedded within ongoing coordinated action. Laughter during demanding movement tasks may also be more reflexive and involuntary (Bryant and Aktipis, 2014; Gervais and Wilson, 2005). In the folk dance sessions, where music and collective movement created particularly challenging acoustic conditions, tactile contact additionally allowed laughter and affective shifts to be physically felt between partners, further reinforcing multimodal coordination.

By contrast, both remote conditions (Zoom and LoLa) fragmented perceptual reciprocity to varying degrees. In the Zoom condition, laughter often remained local and self-directed, failing to circulate across screens and functioning more as a mechanism of self-regulation or explicit verbal negotiation than as shared embodied resonance. The LoLa condition, while still technologically mediated, afforded shared laughter and mutual affective attunement more readily than Zoom, likely due to its lower latency, higher audio–video fidelity, and more immersive setup. Nevertheless, even in LoLa, simultaneous laughter remained less frequent than in the co-located condition, suggesting that subtle disruptions in gaze alignment, breath timing, tactile absence, and micro-temporal coordination continued to affect embodied reciprocity. These findings extend social-functional theories of laughter (Curran et al., 2018; Wood, 2020; Mazzocconi et al., 2021) by suggesting that the affordances of laughter depend not only on social context but also on the degree of multimodal reciprocity available in a communicative ecology.

Methodologically, this study demonstrates the value of combining ecological interaction analysis with multimodal physiological measurements. By analyzing naturally occurring laughter in lived participatory dance settings while simultaneously tracking bodily activity, we were able to examine how laughter operates across physiological, affective, and interactional dimensions simultaneously. Although exploratory and limited in scope, this approach opens new possibilities for studying artistic interaction as a multimodal process of embodied coordination and shared sense-making.

Across the group dancing sessions, laughter was associated with substantial, partially shared increases in heart rate among participants, extending beyond changes attributable to movement intensity alone. Similar patterns of heart-rate fluctuations also appeared in the duo dance sessions, including moments when participants' actions differed considerably. These rapid increases and immediate decreases in heart rate likely reflect the respiratory demands of intense laughter (Bryant and Aktipis, 2014; Gervais and Wilson, 2005), without contradicting the potential for shared laughter to lower heart rates in the long term. The short-term physiological and expressive components of laughter can reorganize collective bodily arousal during socially significant interactions.

Much of the research on the physiological effects of laughter employs prerecorded humorous stimuli such as stand-up comedy sets and funny videos (Averill, 1969; Bains et al., 2017; Law et al., 2018; Schäfer et al., 2025). In contrast, the bouts of laughter in these sessions were rarely detached from participants' immediate embodied involvement. The laughter typically emerged in situations directly tied to the dancers' bodily actions, attempts at coordination, mistakes, uncertainty, negotiation, or social exposure. Participants were not simply reacting to abstract humor or external observation. They laughing in relation to the unfolding demands of coordinating movement together, their present and shared goal in these sessions. This close coupling between laughter, bodily effort, and shared task engagement may help explain the strong physiological resonance observed across participants. In this sense, laughter appeared to align dancers not only affectively and interactionally, but also physiologically, while helping sustain group cohesion during moments of instability or challenge. These findings resonate with evolutionary and social-bonding accounts of laughter and coordinated action (Dunbar, 2022; Tarr and Dunbar, 2024), while also extending these perspectives to the situated, embodied, and interactional dynamics of participatory artistic practice.

Laughter is part of the preparation for the effort to become coordinated, expressing tolerance for creativity and failure, whether or not it is part of the performance. The duo improvisations were performed without laughter; the line dancing group danced to music with only intermittent giggles and cheers; and the folk dancers laughed all the way through their chance to dance together because they were improvising with new partners. The prominence of laughter in these final stages does not determine how laughter was employed to mitigate the challenge of moving around other bodies and to negotiate the progression toward the capacity to perform. Given that laughter has been identified as an important mechanism for social bonding, this study highlights the need to consider the social value of the entire generative process underlying artistic and cultural practices. The final polished form shows only a small part of the effort and rewards participants' experience.

## 6 Conclusions

This exploratory study investigated laughter across co-located, instructional, folk, and telematic dance contexts in order to examine how laughter emerges within participatory movement practices. By combining multimodal interaction analysis with physiological measurements, the study demonstrates that laughter in dance is not merely an emotional by-product of enjoyment or play, but a multimodal coordination resource embedded within collaborative action. Across the cases, laughter repeatedly emerged during moments of uncertainty, negotiation, disruption, effort, and adaptation, helping participants sustain interaction while developing movement together. Rather than interrupting coordination, laughter frequently operated alongside ongoing movement, contributing affective, physiological, and interactional layers to the collaborative organization of dance activity.

The findings further demonstrate that the emergence and circulation of laughter depend strongly on communicative ecology. The spatial, visual, and auditory conditions in the group dance settings were naturally constrained, restricting signaling between participants and with the session instructors. However, acoustically distinctive and multimodal laughter overcame communication barriers, allowing continuous mutual adaptation despite limited sightlines and time for consultation. Laughter also emerged and circulated in the co-located duo dance condition, where auditory, visual, tactile, spatial, and kinaesthetic cues were continuously available to both dancers. In remote settings, where these feedback loops were partially fragmented, laughter circulated less fluidly and became more localized and self-regulatory. These observations extend social-functional theories of laughter by suggesting that the affordances of laughter depend not only on social context but also on the degree of multimodal reciprocity available within a communicative environment.

Physiological measurements substantiated the contribution of dancers' laughter to embodied co-regulation during collaborative activity. Across several cases, laughter was associated with partially shared changes in heart-rate activity that extended beyond movement intensity alone, indicating that laughter may transiently reorganize collective bodily arousal during socially significant moments of interaction. Importantly, these responses emerged not around detached humor but through participants' direct, embodied involvement in the demands of coordinating movement.

Taken together, the findings highlight the broader social significance of the participatory and developmental phases of artistic practices. Laughter was most prominent not during the polished final performance, but during rehearsal, improvisation, experimentation, and the collective work of becoming coordinated together. In this sense, the social value and the bonding effect of dance may reside not only in synchronized movement but also in the shared processes of negotiation, adaptation, repair, and mutual responsiveness through which coordination gradually emerges, often facilitated by laughter.

## Data Availability

Some of the datasets presented in this study can be found in online repositories. Laughter classifications and event annotations for all sessions are included in the analysis github https://github.com/finn42/Laughter_Dance, also available on Zenodo doi: 10.5281/zenodo.20125655. Dancer measurements for the Line dancing lesson are available under Session/LineDancing at the EmbodiedWorkshop2024-dataset repository doi: 10.17605/OSF.IO/RK6QS. Dancer measurements of the Duo Dance sessions and the Folk dancing lesson are not published online but available upon request from the corresponding author according to the terms of sharing specified by participants. Full audio and video recordings of these sessions are not available to protect the anonymity of participants.
